# Variants Located Upstream of *CHRNB4* on Chromosome 15q25.1 Are Associated with Age at Onset of Daily Smoking and Habitual Smoking

**DOI:** 10.1371/journal.pone.0033513

**Published:** 2012-03-16

**Authors:** Manav Kapoor, Jen-Chyong Wang, Sarah Bertelsen, Kathy Bucholz, John P. Budde, Anthony Hinrichs, Arpana Agrawal, Andrew Brooks, David Chorlian, Danielle Dick, Victor Hesselbrock, Tatiana Foroud, John Kramer, Samuel Kuperman, Niklas Manz, John Nurnberger, Bernice Porjesz, John Rice, Jay Tischfield, Xiaoling Xuei, Marc Schuckit, Howard J. Edenberg, Laura J. Bierut, Alison M. Goate

**Affiliations:** 1 Department of Psychiatry, School of Medicine, Washington University, St. Louis, Missouri, United States of America; 2 Department of Genetics, Rutgers University, Piscataway, New Jersey, United States of America; 3 Department of Psychiatry and Behavioral Sciences, SUNY Downstate Medical Center, Brooklyn, New York, United States of America; 4 Department of Psychiatry, Virginia Commonwealth University, Richmond, Virginia, United States of America; 5 Department of Psychiatry, University of Connecticut, Farmington, Connecticut, United States of America; 6 Department of Biochemistry and Molecular Biology, School of Medicine, Indiana University, Indianapolis, Indiana, United States of America; 7 Department of Psychiatry, College of Medicine, University of Iowa, Iowa City, Iowa, United States of America; 8 Department of Psychiatry, University of California San Diego, La Jolla, California, United States of America; Centre for Addiction and Mental Health, Canada

## Abstract

Several genome-wide association and candidate gene studies have linked chromosome 15q24–q25.1 (a region including the *CHRNA5-CHRNA3-CHRNB4* gene cluster) with alcohol dependence, nicotine dependence and smoking-related illnesses such as lung cancer and chronic obstructive pulmonary disease. To further examine the impact of these genes on the development of substance use disorders, we tested whether variants within and flanking the *CHRNA5-CHRNA3-CHRNB4* gene cluster affect the transition to daily smoking (individuals who smoked cigarettes 4 or more days per week) in a cross sectional sample of adolescents and young adults from the COGA (Collaborative Study of the Genetics of Alcoholism) families. Subjects were recruited from families affected with alcoholism (either as a first or second degree relative) and the comparison families. Participants completed the SSAGA interview, a comprehensive assessment of alcohol and other substance use and related behaviors. Using the Quantitative trait disequilibrium test (QTDT) significant association was detected between age at onset of daily smoking and variants located upstream of *CHRNB4*. Multivariate analysis using a Cox proportional hazards model further revealed that these variants significantly predict the age at onset of habitual smoking among daily smokers. These variants were not in high linkage disequilibrium (0.28<r^2^<0.56) with variants that have previously been reported to affect risk for nicotine dependence and smoking related diseases in adults. The data suggests that an age-associated relationship underlies the association of SNPs in *CHRNB4* with onset of chronic smoking behaviors in adolescents and young adults and may improve genetic information that will lead to better prevention and intervention for substance use disorders among adolescents and young adults.

## Introduction

Cigarette smoking is a common addictive disorder. A recent Surgeon General's report indicates that one-third of people who have tried smoking become daily smokers [1]. In 2009, an estimated 20.6% (46.6 million) of Americans aged 18 years or older were current smokers (defined as those who reported that they smoked 100 cigarettes or more during their lifetime and currently smoke every day or some days [2]). Although antismoking campaigns have reduced cigarette use in recent years, the long-term decline of smoking came to a halt in 2010, especially among high school students. In 2010, both 8th and 10th graders showed evidence of an increase in smoking and approximately one in five high school students and adults in the United States continue to smoke regularly [1].

It is well established that the use of tobacco products or exposure to second-hand smoke has detrimental effects on physical health including an increased risk of heart disease, cancer, stroke, and chronic lung disease. In the United States, cigarette smoking accounts for 30% of all cancer deaths and for nearly 80% of deaths from chronic obstructive pulmonary disease [2], [3]. It is also the primary causal factor for early cardiovascular disease and deaths [2].

Many aspects of cigarette smoking behavior, including onset of smoking, smoking persistence, and nicotine dependence, cluster in families [4]. In addition to environmental factors (such as peer pressure), evidence from twin studies indicates that genetic factors contribute to the development of smoking and related addictive behaviors with heritability estimates ranging from 60% to 72% [5]–[7]. Genome wide association studies (GWAS) and candidate gene studies of nicotine dependence have identified several variants in the gene cluster encoding the α5, α3, and β4 nicotinic receptor subunits on chromosome 15 that alter risk for nicotine dependence, including an amino acid substitution (aspartic acid to asparagine at codon 398) in the α5 nicotinic receptor subunit gene (*CHRNA5*) and several non-coding variants [8]–[15]. GWAS using several lung cancer populations have also demonstrated association between lung cancer susceptibility and the same or highly correlated variants in the *CHRNA5-CHRNA3-CHRNB4* gene cluster [16]–[18].

The association of genetic risk factors with nicotine dependence may be influenced by characteristics of smoking behavior such as the number of quit attempts, smoking frequency in adolescents, and age at onset (AAO) of daily smoking [19], [20]. A study using haplotype analysis with variants in the *CHRNA5-CHRNA3-CHRNB4* gene cluster reported that variation in this cluster is associated with severity of nicotine dependence among long-term smokers who began daily smoking at age 16 or younger. Notably, this association was not present among those who began daily smoking after age 16 [21]. To further examine the impact of this gene cluster on the development of nicotine use, we tested whether variants within and flanking the *CHRNA5-CHRNA3-CHRNB4* gene cluster affect AAO of daily smoking in a sample of adolescents and young adults from the COGA (Collaborative Study of the Genetics of Alcoholism) families.

## Materials and Methods

### Study subjects

The Human Studies Committee at the Washington University School of Medicine in Saint Louis approved the study. Approval number for our Collaborative Study on Genetics of Alcoholism: Molecular Biology of Alcoholism is 04-0057. A written informed consent was reviewed and obtained from family members.

The dataset included in this study is a cross sectional sample of the COGA subjects who have had at least one assessment between the years 1989–2008 when they were between the ages of 12 and 25 years. Subjects were recruited from families affected with alcoholism (either as a first or second degree relative) and the comparison families in COGA. The assessment used SSAGA interviews (versions I through 4 and CSSAGA) comprehensive assessments of alcohol and other substance use and related behaviors [22], [23]. The sample consists of 693 families with size ranging from 4–27 (avg. 4.82) and 374 individuals without siblings.

#### Juvenile Diagnoses

All outcome variables related to cigarette use are lifetime measures constructed from each individual's sequential set of direct interviews. The criteria for each of the ever/never measures were examined on each of the interviews in turn and were assessed independently of any other interview(s). Once the criteria for a given measure were met, the variable was coded as present (even if the criteria were not met on a subsequent assessment). The corresponding AAO variable was set to the age reported on and/or computed from the interview on which the subject first met criteria for the lifetime measure.

#### Parental Diagnoses

Parental diagnoses were constructed from each parent's structured set of assessments. This set of assessments consisted of all available direct interviews. However, if there were no direct interviews available, then the sequential set of family-history assessments specific to that parent were substituted. The criteria for each of the ever/never measures were examined on each of the assessments in turn, independently of any other assessment. Once the criteria for a given measure were met, then variable was coded as present and the corresponding AAO variable was set to the age reported.

### Smoking measures

Daily smokers are individuals who smoked cigarettes 4 or more days per week. Age at onset for daily smoking was the AAO from the first interview in which the subject reported daily smoking. Habitual smokers are individuals who smoked at least one pack a day for 6 months or more. Non-habitual smokers are individuals who smoked more than 100 cigarettes lifetime but did not meet the criteria for habitual smoking. Sample characteristics are shown in Table 1.

**Table 1:** Characteristics of study subjects who are daily smokers.

	All Daily Smokers	Habitual Smokers	Non-Habitual Smokers
Number of subjects (% Males)	1312 (53.04)	384 (57.81)	889 (51.29)
European Americans (% Males)	951 (49.94)	324 (55.55)	597 (47.23)
African Americans (% Males)	234 (62.39)	27 (62.96)	201 (62.68)
Unknown ethnicity (% Males)	127 (59.05)	33 (75.75)	91 (52.74)
Mean age at interview (years)	22.04±3.9	23.66±4.26*	21.38±3.44*
Age range at interview (years)	13–38	15–38	14–34
Mean age at onset (years)	16.18±2.62	15.73±2.93	NA
Age range of onset (years)	8–28	8–29	NA

* Indicates t test (two tailed) p = 0.0001.

### Specific SNPs/genes tested

We hypothesized that variants within and flanking the *CHRNA5-CHRNA3-CHRNB4* gene cluster on chromosome 15q24–25.1 region that were associated with nicotine dependence and smoking related phenotypes may also affect AAO of daily/habitual smoking. We tested 10 SNPs (single nucleotide polymorphisms; Table 2) in this gene cluster that tagged variants associated with nicotine dependence and smoking related phenotypes [9], [10]. Genotyping was performed at the Genome Technology Access Center at Washington University School of Medicine in St. Louis (http://gtac.wustl.edu/) using an Illumina GoldenGate custom array as part of a larger set of 384 SNPs.

**Table 2:** QTDT analysis with age at onset of daily smoking as quantitative variable.

	QTDT Total^1^			QTDT Orthogonal^1^		
SNP	N	without rs16969968^2^	with rs16969968^2^	N	without rs16969968^2^	with rs16969968^2^
rs880395	1288	0.02	0.02	212	0.08	0.08
rs7164030	1289	0.02	0.02	214	0.08	0.08
rs16969968	1288	0.25	-	174	0.52	-
rs578776	1289	0.17	0.18	218	0.66	0.66
rs3743078	1289	0.17	0.19	192	0.14	0.13
rs11634351	1289	0.007	0.006	188	0.03	0.04
rs17487514	1288	0.007	0.006	146	0.004	0.005
rs1996371	1288	0.003	0.002	186	0.02	0.03
rs11857532	1287	0.02	0.02	245	0.02	0.02
rs922692	1288	0.003	0.003	183	0.02	0.03

1 Age at interview in quartiles, gender, first principal component of stratification (pc1), parental smoking and parental drinking were included as covariates.

2 Nominal P values are shown uncorrected for multiple testing. The Bonferroni-corrected significance threshold is P = 0.005.

### Data analysis

Association analyses with age at onset of daily smoking in daily smokers were conducted using the statistical software QTDT (Version 2.4.5, http: sph.umich.edu/csg/abecasis/QTDT; [24]). Among daily smokers both total and within-family (orthogonal model) evidence for association was assessed using age at onset of daily smoking as a continuous variable. The orthogonal model is the generalization of the Fulker model [24], allowing for families of any size (with or without parental genotypes) and was used in a variance components framework. QTDT total association test evaluates total evidence for association and is more powerful than QTDT orthogonal test in absence of population stratification. Therefore we first evaluated the evidence of population stratification by comparing the between and within components of association using QTDT stratification test.

The age at onset for habitual smoking among daily smokers was analyzed using Cox proportional hazards regression analysis (coxph) using the R statistical analysis package. This analysis can incorporate a clustered sandwich estimator to account for the familial correlation among observations. The age at onset measures for non-daily smokers were right-censored at each subject's age at their most recent assessment. The first principal component from the stratification analysis (pc1), age at interview, gender, maternal/paternal daily smoking and maternal/paternal regular drinking were included as covariates. The violation of the proportional hazards assumption was tested with non-zero slope of Schoenfeld residuals versus time using the survival analysis package in R.

Previous studies have demonstrated that a non-synonymous coding SNP, rs16969968 in *CHRNA5*, increases the risk for nicotine dependence [8]–[15]. To test whether rs16969968 underlies the association between variants upstream of *CHRNB4* and AAO of daily smoking, we included rs16969968 as a covariate in QTDT and survival analysis.

## Results

### Variants located upstream of CHRNB4 are significantly associated with age at onset of daily smoking using QTDT analysis

In this data set, QTDT finds no evidence for stratification therefore we performed QTDT-total test to evaluate the total evidence for association. Several SNPs show significant association with AAO of daily smoking (Table 2). The most significantly associated variant is rs1996371, located upstream of the *CHRNB4* gene (p = 0.002). Individuals homozygous for the risk allele had AAO at least one year earlier than individuals who are homozygous for the non-risk allele; the difference in AAO between these two groups was statistically significant (p = 0.0002; Table 3). Variants rs11634351 and rs922692 that are highly correlated with rs1996371 (r2 = 0.98 and r2 = 0.95, respectively) also show association with AAO of daily smoking. Conditional analysis showed that association of rs1996371 and correlated SNPs with AAO of daily smoking is independent of rs16969968 (Table 2). Conditional analysis using variants rs578776 and rs880395, which represent other distinct loci in this gene cluster produced similar results and confirmed the independent relationship of rs1996371 (p<0.005). Variants rs1996371 and rs922692 withstood the conservative Bonferroni correction (<0.005). Because most of the markers used in this study are highly correlated we also performed the less conservative correction of SNPs in LD with each other given by Nyholt et al [25]. This method provides the effective number of independent marker loci and in the present study this number was 5, giving an experiment-wide significance threshold of p<0.01. According to this less stringent criterion for correction, rs1996371, rs11634351, and rs922692 were significant after correction for multiple testing.

**Table 3:** Distribution of age at onset of daily smokers with reference to alleles of rs1996371.

Genotype	Daily Smoker	Habitual Smoker	Non-Habitual Smoker
	Mean age at onset (N)	Mean age at onset (N)	Mean age censored (N)
AA	16.41±2.6 (611)*	15.67±3.09 (147)	21.54±3.45 (465)
AG	16.07±2.6 (518)	15.82±2.92 (173)	21.15±3.40 (330)
GG	15.56±2.4 (162)*	15.55±2.59 (64)	21.42±3.48 (94)

* Indicates t test (two tailed) p = 0.0002.

### Variant rs1996371 and highly correlated SNPs affect age at onset among daily smokers using survival analysis

**Figure 1:**
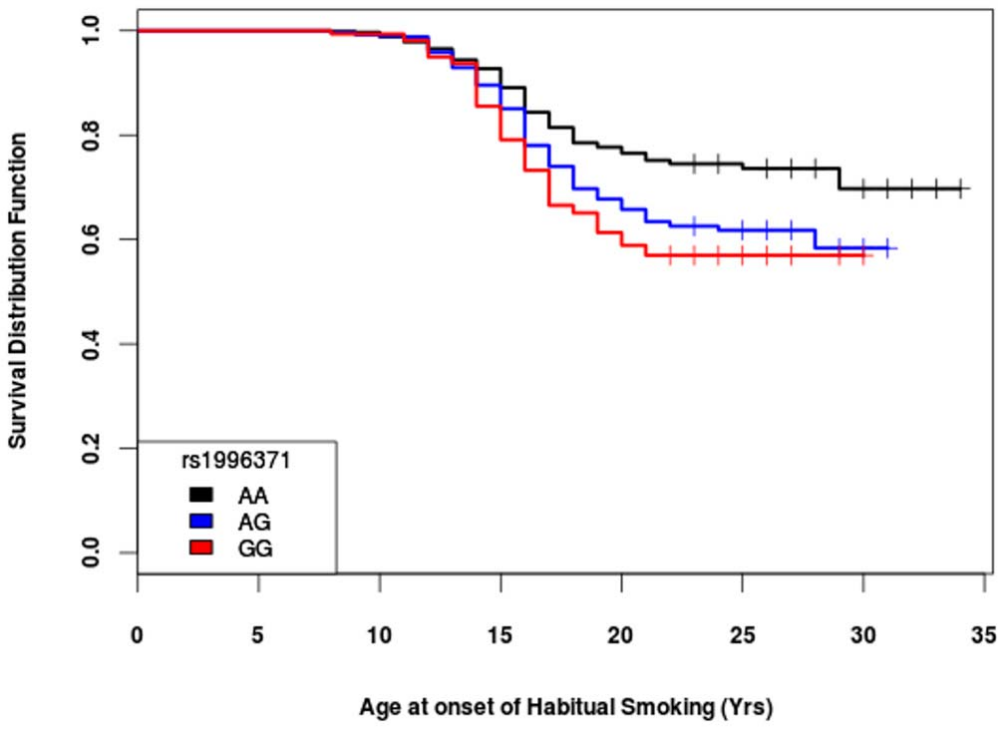
Genotypes of rs1996371 significantly predict age at onset of habitual smoking.

We further explored the effects of rs1996371 on smoking behaviors by testing whether this SNP modifies the AAO of habitual smoking among subjects who are already daily smokers. For non-habitual smokers, the AAO measures were right-censored at each subject's age at their most recent assessment. Our Cox-proportional analysis using AAO of habitual smoking censored at non-habitual smokers demonstrated that the individuals who have a minor allele of rs1996371 and highly correlated variants have 1.2 times more chance of becoming habitual smokers with every one-year increase in their age (Table 4). We tested the proportional hazard assumption for all covariates using Schoenfeld residuals and no violation was detected. The strongest effect of association with rs1996371 is between 14 and 21 years (Figure 1). At age 21, 43% of individuals with 2 copies of the minor allele of rs1996371 are habitual smokers while 25% of individuals homozygous for the major allele are habitual smokers (Figure 1). We also performed the same analysis using rs16969968 as a covariate and confirmed that the effect of rs1996371 and correlated variants on AAO of habitual smoking is independent of the influence of rs16969968 (Table 4). The effect remained significant after controlling for other variants in this gene cluster (HR≃ 1.23, P<0.005) and Bonferroni correction for multiple testing. Some earlier studies showed that gender is a major factor influencing smoking behaviors. In our stratified analysis, although power was reduced, rs1996371 and correlated SNPs significantly predicted risk for habitual smoking with increasing age in females and males (females: rs1996371, p = 0.05, HR: 1.25; males: rs1996371, p = 0.02, HR:1.2).

**Table 4:** Cox-proportional hazard analysis using age at onset of habitual smoking censored at non-habitual smokers.

	without rs16969968 as a covariate^1^			with rs16969968 as a covariate^1^		
Marker	N	Hazard ratio (95%CI)	p value^2^	N	Hazard ratio (95%CI)	p value^2^
rs11634351	1273	1.23 (1.07–1.43)	0.005	1288	1.23 (1.05–1.44)	0.008
rs17487514	1273	1.14 (0.97–1.34)	0.11	1288	1.13 (0.95–1.35)	0.15
rs1996371	1272	1.24 (1.07–1.43)	0.004	1288	1.23 (1.05–1.44)	0.008
rs11857532	1271	1.14 (0.99–1.32)	0.05	1288	1.14 (0.98–1.32)	0.09
rs922692	1272	1.24 (1.07–1.43)	0.004	1288	1.23 (1.06–1.44)	0.008

1 Analysis was performed within family clusters using additive model. Age at interview in quartiles, gender, pc1, parental smoking and parental drinking were included as covariates.

2 Nominal P values are shown uncorrected for multiple testing. The Bonferroni-corrected significance threshold is P = 0.005.

## Discussion

People who start smoking at a young age are more likely to have higher lifetime amounts of tobacco smoked and consequently are at greater risk of becoming nicotine dependent [19]. Variants flanking and within the *CHRNA5-CHRNA3-CHRNB4* gene cluster region affect the risk for nicotine dependence [8]–[15], and are also involved in the transition toward heavy smoking in mid-adulthood and in smoking persistence [26]. Studies using haplotype analysis reported that common haplotypes in this gene cluster are associated with nicotine dependence in adults especially among those who began daily smoking before age 16 [21]. These haplotypes also linked to nicotine withdrawal and smoking cessation phenotypes though the associations were not influenced by AAO of daily smoking [27]. In this study, we conducted family-based association tests using AAO of daily smoking as a quantitative phenotype. We identified an association between AAO of daily smoking and a group of correlated variants located upstream of *CHRNB4* gene in a cohort of young adults, that was independent of the effect of rs16969968.

**Figure 2:**
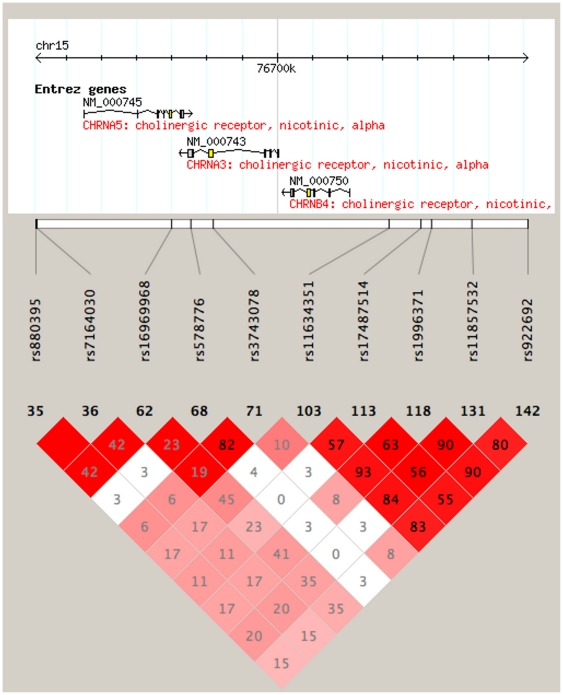
Linkage disequilibrium between genotyped SNPs. Number in each square represents the a pairwise LD relationship (r^2^) between the two SNP's in Caucasians using HapMap data and varying red color represent the linkage disequilibrium values for that pair as measured by D′ (bright red shows high D′).

There are many environmental factors such as influence of peer group [28], lack of parental monitoring [29] and easy access to cigarettes can also influence the genetic liability to smoking behavior in young adults. Koopmans et al [30] reported that the total variance accounted by genetic influences on smoking initiation and cigarettes per day is 39% and 86%, respectively. The liability to ever smoking is largely independent from liability to continue to smoke. This could be due to the fact that while over 80% of individuals in some studies report that they have tried smoking cigarettes, only about one third of them will smoke beyond experimentation [31]. Hence genetic factors may have stronger impact on daily smoking (smoked ≥4 days/week) and habitual smoking (one pack a day for 6 months) rather than smoking initiation. Therefore in the present study we focused on subjects ascertained as daily smokers to find out the relationship between *CHRNA5-CHRNA3-CHRNB4* variants and onset of daily/habitual smoking behaviors, believing that this selected group can explain more genetic variance than smoking initiation or exposure. We found that the rs1996371 and correlated variants located upstream of *CHRNB4* gene were associated with age at onset of daily smoking. Since our sample is multi-ethnic we also restricted our analyses to the largest subgroup (European Americans) to ensure that the association was not a false positive due to heterogeneity in our dataset. The association remained in this subgroup. The association between age at onset of daily smoking and rs1996371 and correlated SNPs also remained statistically significant after correcting for multiple testing proposed by Nyholt et al [25], (rs1996371, p = 0.0058). The daily smokers who had at least one copy of the minor allele of rs1996371 had a mean AAO of daily smoking 1 year younger than individuals without the minor allele. The non-synonymous SNP, rs16969968 was not associated with age at onset of daily smoking. We further wanted to explore whether daily smokers with these variants are at increased risk of becoming habitual smokers in future. Our Cox-proportional hazard analysis indeed showed that smokers who have an early AAO of daily smoking and carry a minor allele of rs1996371 have approximately 1.2 times higher risk of becoming habitual smokers compared to daily smokers who carry the major allele. This association was further confirmed in stratified analysis in EAs (rs1996371, p = 0.03, HR: 1.17). In African Americans the variant was not statistically significant, most likely because of low power, but the effect was in the same direction (rs1996371, p>0.05, HR: 1.21). SNP rs1996371 has low correlation (0.28<r^2^<0.56 in Europeans using HapMap data, Figure 2) with variants (e.g. rs1051730, rs16969968) that were reported to affect risk for nicotine dependence and smoking related diseases in adults. Interestingly Schlaepfer et al reported a variant in the 3′UTR of *CHRNB4* gene, rs1948, has been linked to early age of initiation for tobacco use in an ethnically diverse young adult cohort [32]. They also found that a variant, rs11634351 that is in low LD with rs1948 (r2 = 0.24) had significant association with age at first drink [32]. In current study we found that rs11634351 is associated with AAO of daily smoking and is in complete LD with rs1996371 (Figure 2). High comorbidity among smoking and drinking behaviors might explain this possible overlap [33].

The most significant variant in this study (rs1996371) was also found to be associated with *CHRNB4* mRNA expression in human brain (p = 0.01) [34]. There are a few functional studies that have reported the possible involvement of the *CHRNB4* gene with drugs of abuse. Bruschweiler-Li et al. demonstrated that a 2.3-kb fragment of the *CHRNB4* promoter region containing the CA box is capable of directing cell-type specific and developmentally regulated expression of a reporter gene *in vivo* [35]. There is also some indication that blockade of α3β4 nAChRs results in a reduction of opioid and stimulant self-administration, suggesting that nAChRs that contain the β4 subunit are involved in mediating withdrawal syndromes elicited by some drugs of abuse [36]. In a study using transgenic mice, Frahm and colleagues [37] showed that targeted overexpression of β4 leads to strong aversion to nicotine. This study further provided evidence that the medial habenula acts as a gate-keeper in the control of nicotine consumption and that the balanced contribution of β4 and α5 subunits is critical for this function. Thus, further studies elucidating the molecular mechanisms underlying expression of the β4 gene may improve our understanding of nicotine addiction and withdrawal as well as lung cancer, and other smoking-related diseases.

In this study we identified distinct variants in the *CHRNA5-CHRNA3-CHRNB4* gene cluster that influence the age at onset of habitual smoking in young adults. These variants are not in LD with rs16969968 or its correlates that are associated with nicotine dependence. Earlier Schlaepfer and coworkers [32] also showed that variants other than rs16969968 or its correlates affect age at initiation of tobacco use at early age. These results show that there may be different mechanisms, which promote smoking behaviors at younger ages. The age dependent interplay of nicotinic receptors subtypes could explain the process of initiation to persistence of chronic smoking behaviors, but there remains a need for follow-up studies to confirm the hypothesis.
